# High Epitope Expression Levels Increase Competition between T Cells

**DOI:** 10.1371/journal.pcbi.0020109

**Published:** 2006-08-18

**Authors:** Almut Scherer, Marcel Salathé, Sebastian Bonhoeffer

**Affiliations:** Theoretical Biology, Institute of Integrative Biology, Swiss Federal Institute of Technology, Zurich, Switzerland; University of New South Wales, Australia

## Abstract

Both theoretical predictions and experimental findings suggest that T cell populations can compete with each other. There is some debate on whether T cells compete for aspecific stimuli, such as access to the surface on antigen-presenting cells (APCs) or for specific stimuli, such as their cognate epitope ligand. We have developed an individual-based computer simulation model to study T cell competition. Our model shows that the expression level of foreign epitopes *per APC* determines whether T cell competition is mainly for specific or aspecific stimuli. Under low epitope expression, competition is mainly for the specific epitope stimuli, and, hence, different epitope-specific T cell populations coexist readily. However, if epitope expression levels are high, aspecific competition becomes more important. Such *between*-specificity competition can lead to competitive exclusion between different epitope-specific T cell populations. Our model allows us to delineate the circumstances that facilitate coexistence of T cells of different epitope specificity. Understanding mechanisms of T cell coexistence has important practical implications for immune therapies that require a broad immune response.

## Synopsis

Pathogens are masters of disguise, and frequently escape recognition by the immune response. Therefore, broad immune responses, directed at many epitopes of the pathogen, are thought to improve control of infection. There is evidence that competition between immune cells of different epitope specificity reduces the breadth of the immune response. It has been suggested that the resource that T cells compete for is access to antigen-presenting cells (APCs). However, the experimental data regarding competition for access to APCs is controversial. In this study, Scherer, Salathé, and Bonhoeffer have used an individual-based model to investigate the mechanisms of T cell competition. They find that T cells only compete for access to APCs when epitopes are expressed abundantly on APCs. In contrast, when epitope expression is limiting, competition is for the specific epitope rather than for access to APCs. The distinction between competition for epitope and for access to APCs is relevant because the model predicts qualitatively different outcomes for either case. When competition is for the specific epitope, different epitope-specific T cell responses coexist readily and hence the immune response is broad. However, when T cells compete for access to APCs, immunodominant T cell responses can outcompete subdominant ones, which leads to narrow immune responses.

## Introduction

From an ecologist's perspective, the maintenance of a broad immune response, simultaneously recognising multiple antigenic regions of a pathogen, is puzzling. Ecological theory predicts that the number of coexisting species in a certain place and time cannot exceed the number of limiting resources in the system [1]. To avoid competition, species are thought to differentiate into their niche, which is their specific set of resources they depend on to survive and reproduce. The lower the resource differentiation between species, the smaller is their niche overlap, and the weaker are the competitive interactions between them. As in ecosystems, cells of the immune system depend on limiting resources. T cells, one of the main types of immune cells of the adaptive immune system, proliferate in response to antigen stimuli, and they might therefore compete for these stimuli. Yet, upon infection of a host, T cell populations specific for many parts of the pathogen tend to be triggered. What resource differentiation mechanisms might allow the immune response against a pathogen to be diverse? In this paper we use a stochastic computer simulation model to investigate this question.

When a pathogen infects a vertebrate host, it is displayed to the immune system on antigen-presenting cells (APCs) as epitopes (eight-to-ten-amino-acid-long protein fragments), bound to major histocompatibility complex (MHC) class I molecules. T cells specific for any of the displayed epitopes can become activated, upon which they proliferate rapidly and clear pathogen-infected cells. Most effector T cells die rapidly after the expansion phase of the response, unless they reencounter remaining foreign antigen in the lymphoid tissue. T cell populations specific for different epitopes of a pathogen depend on a shared pathogen resource for their expansion, and might therefore compete for this resource. Nevertheless, coexistence of broad T cell responses, targeting several or many epitopes of the pathogen, seems to be the rule rather than the exception, both in acute infections [2–4] and in chronic infections [5–8]. The functional importance of broad immune responses is thought to be related to immune escape, that is, the acquisition of mutations in the pathogen that abrogate immune recognition. Therefore, broad responses are thought to improve control of infections. The mechanisms that influence the breadth and immunodominance (i.e., size hierarchy of the different epitope-specific T cell populations) of a T cell response are manifold, and include differences in the T cell precursor frequency for a certain epitope [9], epitope expression levels [10–12], and competition between T cells [13]. This paper focuses on the last factor and asks under which conditions T cells of different epitope specificity compete with each other.

There is a considerable body of experimental data on T cell competition, recently reviewed by Lanziavecchia, et al. [14], that forms the basis of our current understanding of its mechanistic basis. We here restrict ourselves to describing examples of competition data for cytotoxic T cells (CD8+ T cells), since these are the focus of our model. However, data for competition between helper T cells (CD4+ T cells) also exists (e.g., [15]). T cell competition firstly only occurs if the resource, that is, the epitope displaying APCs, is limiting. Hence T cell competition can only be observed when the ratio of T cells to antigen is high [16,17]. Second, competition between T cells of different epitope specificity is related to competition for access to the surface of APCs, since presenting epitopes on separate APCs abrogates competition [18]. The current understanding of T cell competition is largely based on adoptive transfer experiments and on epitope knock-out studies. In adoptive transfer experiments, T cells specific for a particular epitope are transferred into a mouse, and subsequently, the mouse is immunised with the appropriate epitopes. A consistent result of adoptive transfer experiments is that the transferred T cells interfere with the host T cell response of the same epitope specificity (within-specificity competition). However, the evidence regarding competition between T cells of different epitope specificity (between-specificity competition) is controversial. In Kedl, et al. [18], the expansion of host T cells against epitopes unrelated to that of the transferred T cell specificity was reduced by the adoptive transfer, while in Probst, et al. [19] it was not. This between-specificity competition was interpreted as competition for access to APCs. An analysis of in vitro competition data with a mathematical model also yielded evidence for competition for access to APCs [20]. In epitope knock-out studies, one or several dominant epitope-specific responses are reduced or inhibited fully, either by interfering with presentation of the viral epitopes [21,22] or by inducing thymic tolerance against them [23]. This inhibition of dominant responses led to an increase of the size of subdominant ones. Such compensation effects were typically seen in secondary challenges, when the ratio of T cells to antigen is high. However, in an epitope knock-out experiment with a bacterial pathogen, the response to subdominant epitopes was unaltered by the removal of one or even two dominant epitopes from the bacterial pathogen [24]. The reason for this discrepancy between compensation effects in viral and bacterial infections is not clear.

Most previous modelling approaches on T cell dynamics and T cell competition describe the interactions between T cells and infectious agents in a predator–prey-like manner [25,26], where T cells corresponded to the predators and the epitope-presenting APCs corresponded to the prey. APCs are often modelled as independent T cell interaction sites on APCs that are either free or engaged in an interaction with a T cell [25–27]. *Independent* in this context means that the interaction of a T cell with one APC site does not affect the status of other sites on the same APC. These sites are further assumed to present sufficient amounts of epitope for each of the competing T cell populations, and competition for access to these APC sites leads to competitive exclusion of the subdominant T cell specificity. Several modelling approaches have been made to allow for coexistence of multiple epitope-specific T cell populations, but in few is coexistence based on mechanistic assumptions. One study modelled the expansion dynamics of T cells heuristically, by feeding observed expansion and contraction dynamics into the model [28]. Another assumed a heuristic term of competition between T cells of the same epitope specificity [29]. This increased within-specificity competition implies a reduced niche overlap between T cells of different epitope specificity and hence allowed for multiple epitope-specific T cell responses to coexist. The assumed basis for the higher intensity of within-specificity competition than of between-specificity competition suggested by Korthals-Altes, et al. [29] was that epitope-specific T cell populations might expand locally in tissue, and hence interact more with each other than with cells of other specificities. Finally, we have recently developed a model in which the interaction between T cells and APC sites leads to down-modulation of the specific epitope. This assumption was based on experimental evidence [30], and led to resource differentiation on APC sites, thus allowing for coexistence of multiple epitope-specific T cell populations [31]. A lot of the previous modelling work on T cell competition has been done in the form of ordinary differential equations. The benefit of using ordinary differential equation models lies in their simplicity, which allows some results to be derived analytically. However, ordinary differential equations are also less flexible in terms of incorporating biological features of the modelled organisms. In this paper, we develop an individual-based model (IBM) to study potential mechanisms for coexistence of multiple epitope-specific T cell populations. In particular, using an IBM allows us to model APCs with multiple, interconnected T cell interaction sites. This is in contrast to previous modelling in which APC sites were considered as independent entities.

Our main finding is that, at low per APC epitope expression levels, T cell competition is specific, namely for access to the specific epitope ligand. Therefore, T cells of different epitope specificity do not interfere with each others' expansion, and diverse T cell responses can coexist. However, at high epitope expression levels, competition becomes aspecific, namely for access to the surface of the APC, resulting in competitive exclusion. Our results suggest that the epitope expression level per APC might critically affect T cell competition and coexistence. This effect can only be observed when T cell interaction sites on APCs are interconnected, as is the case in our IBM.

## Results

Our simulation describes the dynamics of virally infected cells, APCs, and cytotoxic T cell populations specific for different epitopes of the virus. In Figure 1 the central features of the model are illustrated, and in the Materials and Methods section the model is described in detail and the parameter values are listed.

**Figure 1:**
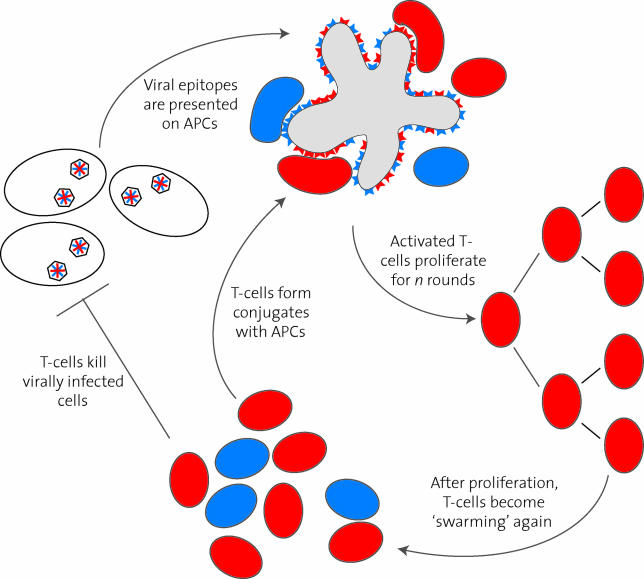
The Basic Features of the IBM The production of APCs depends on the number of virally infected cells. APCs express two to *n* different pathogen epitopes. We refer to the infecting pathogen as a virus in this paper, but the model applies to all intracellular pathogens and extracellular pathogens that enter the MHC class I presentation pathway via cross-presentation [78,79]. For simplicity, APCs present the same amount of each epitope, though in the discussion of this paper, we discuss the effect of skewed epitope expression levels. Epitopes are assumed to be able to move freely in the membrane of the APC, and when a T cell forms a conjugate with a site on an APC, the amount of epitope the T cell requires for activation is allocated into the site of T cell:APC interaction. Unless stated otherwise, each APC is assumed to have six T cell binding sites. In the illustration, each blue and red crown-like symbol on the surface of an APC represents ten copies of epitope for which blue- and red-coloured T cells, respectively, are specific. T cells are assumed to require 50 copies of their cognate epitope to become activated. Upon dissociation of the conjugate, T cells become activated with a probability set by the probability of proliferation of the T cell (“T cell affinity”). Upon activation, T cells go through *n* rounds of programmed proliferation (*n* is set to five unless stated otherwise), after which they become effector T cells and clear virally infected cells. We assume that T cells spend some time in infected tissue before reentering the blood circulation and homing back to lymphoid tissue. Hence, scanning of APCs by T cells recommences after an average of five days after proliferation. Until they form a new conjugate with an APC, they perform their effector function and kill virally infected cells. T cells and APCs die with a probability of 0.2 per day. A more detailed description of the model and of the parameter values used in the model can be found under Materials and Methods.

### Less Is More: T Cell Coexistence Depends on Epitope Expression Levels

In Figure 2, we show simulations of the dynamics of two T cell populations specific for two epitopes of a virus in acute (Figure 2A and 2B) and chronic (Figure 2C and 2D) infection, for either high (Figure 2A and 2C) or low (Figure 2B and 2D) per-APC expression levels of the epitopes. When epitope expression levels are high, the subdominant T cell population expands very poorly, and is eventually outcompeted by the immunodominant one, in both acute and chronic infection. In contrast, at low epitope expression levels, both responses expand well and coexist. Why do T cells of different epitope specificity interfere more with each other when epitopes are expressed at higher densities on APCs?

**Figure 2:**
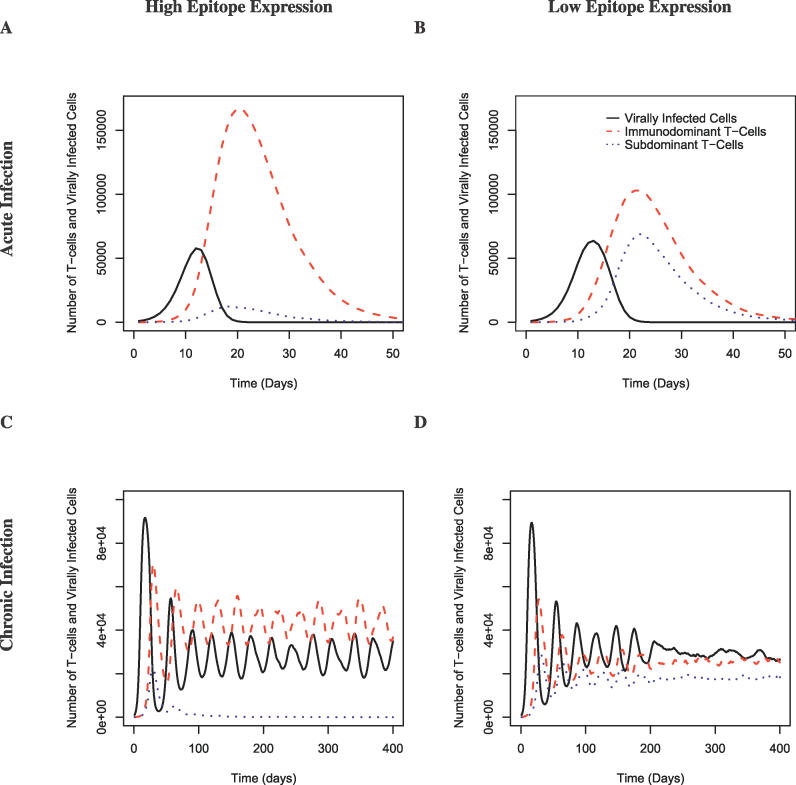
T cell Dynamics in Acute and Chronic Infection In these simulations, two epitopes of a pathogen are displayed at equal densities on APCs. The number of epitope copies required for T cell activation is set to 50 copies, and APCs have six T cell binding sites. (A and C) Both epitopes are displayed at high expression levels of 300 copies each (per APC). (B and D) Both epitopes are displayed at low expression levels of 150 copies each. Acute infection is modelled by assuming a higher proliferative capacity of T cells in acute than in chronic infection, namely seven versus five rounds of proliferation, respectively. T cell affinities are set to 0.07 and 0.1 for the subdominant and immunodominant T cell specificity, respectively. All other parameter settings are listed in Table 1.

**Table 1:**
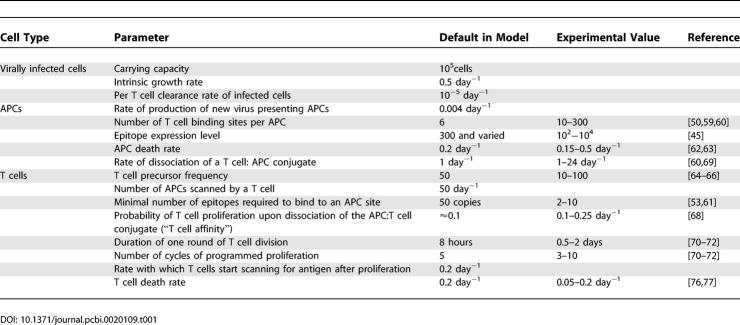
Parameter Values of the Model and Experimental Estimates

To address this question, we follow T cell coexistence, defined as the ratio of the subdominant versus the immunodominant immune response size after 400 days of chronic infection, as a function of the per-APC epitope expression. Figure 3 reveals three qualitatively different regimes of T cell competition: (i) only specific competition, (ii) both specific and aspecific competition, and (iii) only aspecific competition. In the following, we discuss in more detail how epitope expression levels shape each of these competition regimes.

**Figure 3:**
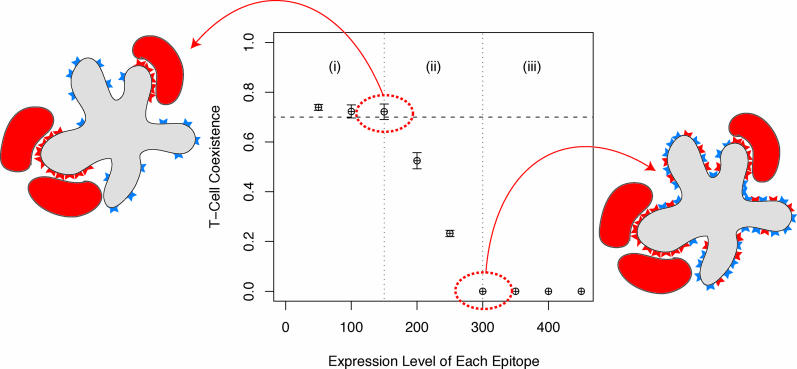
The Effect of the per-APC Epitope Expression Level on T Cell Coexistence Shown is the mean ± standard deviation of three replicate simulations. The black dashed line indicates the ratio of epitope affinities of the subdominant and the immunodominant T cell specificity, which were set to 0.07 and 0.1, respectively. All other parameter settings are listed in Table 1. Left and right of the plot are illustrations of an APC with low (150 copies) and high (300 copies) per-APC epitope expression levels, respectively. In the illustrations, three T cells of the immunodominant specificity are conjugated with the APC. For low epitope expression levels this leads to a situation in which the remaining space on the APC only presents the subdominant epitope, while for high epitope expression levels the remaining space presents both epitope specificities.


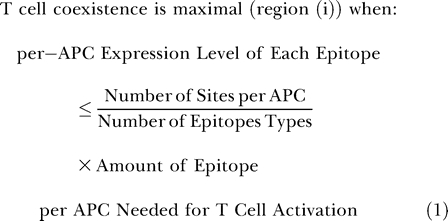


For the parameter settings used so far, where a T cell requires 50 copies of epitopes to be activated and APCs have six T cell binding sites, Equation 1 shows that competition is for the specific epitope for epitope expression levels up to 150 copies of each epitope per APC. The diagram at the left of Figure 3 illustrates why T cells of different epitope specificity do not compete for each other at such low epitope expression levels. When a T cell forms a conjugate with an APC, the amount of specific epitope that the T cell requires for activation is allocated to that site and is thus inaccessible for other cells. If for example three T cells specific for the immunodominant epitope have formed conjugates with an APC that expresses 150 copies of each epitope, the remaining three sites will solely present the subdominant epitope. Hence, under such limiting epitope expression levels, T cells of different epitope specificity do not compete with each other directly, and the level of T cell coexistence (i.e., the ratio of subdominant to immunodominant population size) is given by their relative T cell affinities.


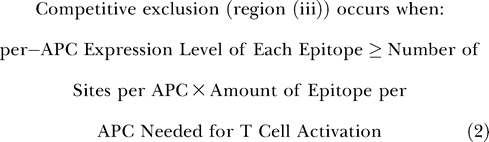


For epitope expression levels above 300 copies of each specificity (region (iii)), all T cell binding sites of the APCs present sufficient copies of each epitope specificity to allow T cells to form a conjugate. At such high epitope expression levels, the limiting factor is no longer epitope, but rather access to T cell binding sites on APCs. In this situation, both T cell specificities depend on the same resource, i.e., competition within and between T cell specificities is equivalent, and the immunodominant T cell specificity outcompetes the subdominant one because it replicates faster (higher probability of proliferation). Therefore, T cell coexistence is lost at high epitope expression levels.

For epitope expression levels ranging from 150 to 300 copies of each specificity (region (ii)), some APC sites only express enough of one epitope specificity, while others express enough of both specificities. This means that there is both shared and unshared resource for the two T cell populations. The more APC sites express sufficient epitope of both specificities, the higher the competition between T cells of different epitope specificity, and consequently, the lower the coexistence of the two T cell specificities.

In Figure 3, the relative affinity of the subdominant versus the immunodominant T cell specificity was 0.7. We next investigated whether the range of affinities of T cells that can coexist is limited to very similar affinities, or if T cells of quite distinct affinities can coexist. In Figure 4, T cell coexistence is plotted over a wide range of relative affinities of the subdominant versus the immunodominant specificity. The black line in Figure 4 represents simulations with high epitope expression levels (300 copies of each epitope), the red line represents medium epitope expression levels (200 copies of each epitope), and the blue line represents low epitope expression levels (150 copies of each epitope). Since resource differentiation is complete at this low epitope expression level of 150 copies of each epitope, the ratio of the two T cell populations (the T cell coexistence in Figure 4) equals the relative affinity of the subdominant versus the immunodominant T cell. Therefore, the slope of T cell coexistence versus relative T cell affinity at low epitope expression levels equals one. At very low affinity of the subdominant T cell specificity, coexistence is lost, both at low and medium epitope expression level. This is because, at low epitope affinity, the subdominant T cell population divides too rarely to compensate for the death rate of the T cells.

**Figure 4:**
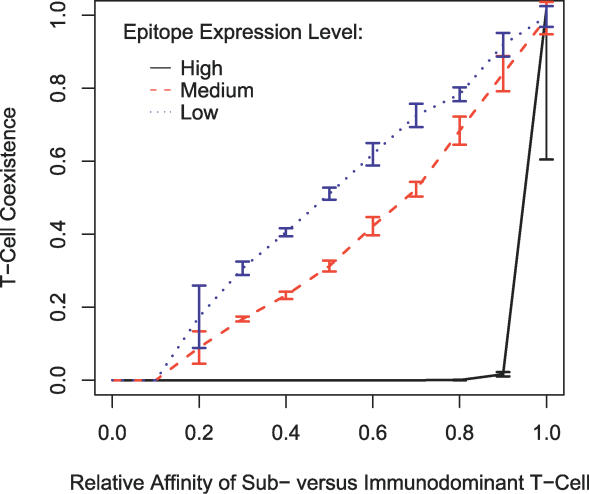
T cell Coexistence Depends on the Relative Affinity of the Immunodominant versus the Subdominant T Cell In the simulations for this plot, the affinity of the immunodominant T cell specificity is set to 0.1, and the affinity of the subdominant is increased from zero to 0.1. The black, red, and blue lines are for high, medium, and low epitope expression levels of 300, 200, and 150 copies of each epitope, respectively. Shown is the mean ± standard deviation of six replicate simulations. All other parameter settings are listed in Table 1.

### The Surface Area of APCs Sets the Maximal Breadth of an Immune Response

Up to this point we have studied the question of competition and coexistence of multiple epitope-specific T cell responses in the simplest setting of two epitopes and two T cell populations. In this section, we investigate T cell coexistence in a model with many epitopes, and ask whether there is a limit to the number of T cell populations that can coexist in equilibrium. To address this question, we simulate our model with APCs that have six or ten T cell binding sites (Figure 5A and 5B versus Figure 5C and 5D, respectively). When epitopes are expressed at exactly the amount required to activate a T cell (i.e., 50 copies), essentially all presented epitopes are targeted by a T cell response. If epitope expression levels are increased above the minimal amount needed for T cell activation (i.e., for epitope expression levels of 100 copies and higher), epitope-specific responses are lost sequentially, in order of their affinity ranking. Interestingly, for this range of epitope expression levels, the diversity profiles of T cell responses generated with six-site APCs presenting six or ten epitopes are indistinguishable. Similar results were obtained with APCs with ten T cell binding sites. For these, the profiles of T cell response diversity of APCs presenting 10 and 17 epitopes were equivalent for all but the epitope expression level of 50 copies per epitope per APC. Again, at this level, essentially all presented epitopes were targeted (Figure 5C and 5D).

**Figure 5:**
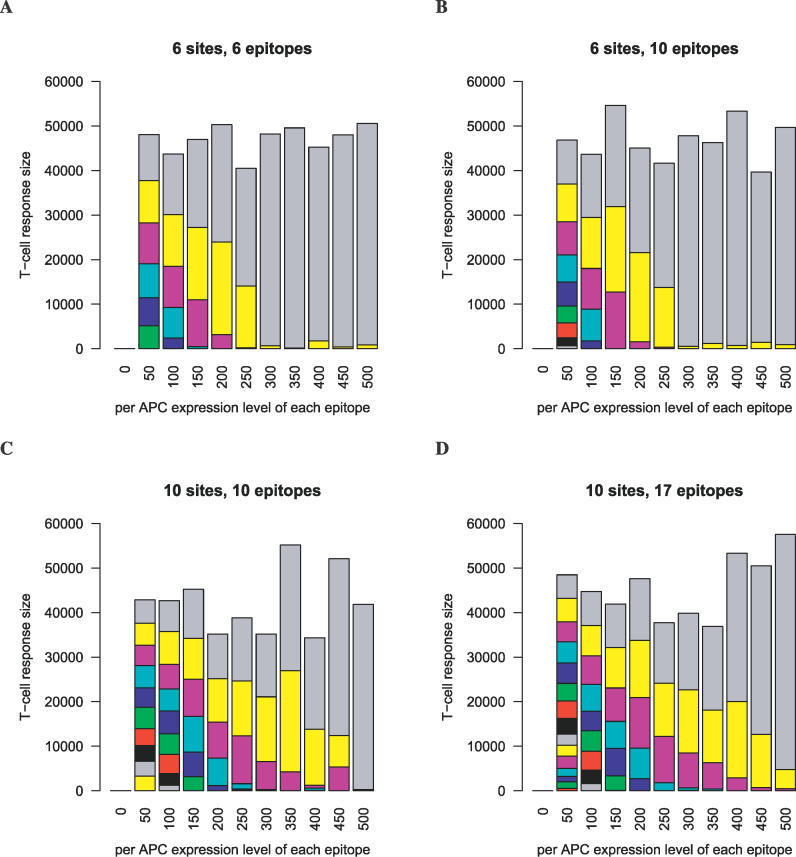
T Cell Diversity Decreases with Increasing per-APC Epitope Expression Level APCs with six T cell binding sites were simulated with six or ten presented epitopes (A and B, respectively), and APCs with ten T cell binding sites with 10 and 17 epitopes (C and D, respectively). Per bar, the different epitope-specific T cell populations are stacked and represented in different colours. For increasing epitope expression levels, T cell diversity drops from its maximum, which is when epitopes are presented at the amount necessary to activate a T cell (i.e., 50 copies of epitope), down to one for high epitope expression levels. Diversity reaches one when each epitope is expressed at sufficient levels to activate a T cell of the corresponding epitope specificity at *each* site of the APC, which is at epitope expression levels of 300 and 500 for APCs with six and ten T cell binding sites, respectively. T cell affinities of epitopes presented on APCs with six sites were as follows: for six epitopes, [0.05, 0.06, 0.07, 0.08, 0.09, 0.1], and for ten epitopes [0.03, 0.035, 0.04, 0.045, 0.05, 0.06, 0.07, 0.08, 0.09, 0.1]. On APCs with 10 sites and 10 epitopes, affinities ranged from 0.0375 to 0.06, and on APCs with 10 sites and 17 epitopes, affinities ranged from 0.02 to 0.06. Subsequently ranked T cell specificities always differed by 0.0025 in terms of their affinities. All other parameter settings are listed in Table 1.

These data suggest that when the total number of epitope types presented on an APC is larger than the available space in terms of T cell binding sites some epitopes will not elicit an immune response, even if epitopes are expressed at very low levels. In the special case when epitope presentation equals the amount a T cell requires for activation, the number of coexisting T cell specificities can exceed the number of T cell binding sites on the APC. For example, on an APC with ten T cell binding sites that presents 17 epitope types at 50 copies each, 15 epitopes were targeted by the immune response. This is because as soon as a T cell of a certain specificity has formed a conjugate with the APC, all other sites cannot stimulate T cells of the same specificity anymore. Hence, the within-specificity competition is much stronger than the between-specificity competition.

### Epitope Expression Levels in Infections

Broad immune responses are thought to be important to protect infected hosts against rapidly evolving pathogens that can accumulate mutations that interfere with presentation of the epitope or recognition of the epitope by the specific T cells. Loss of an immune response due to such mutations is called immune escape, and is thought to be a central problem of the immune system's fight against HIV [32–37].

Our model suggests that the breadth of T cell immune responses is maximal when the per APC epitope expression levels are close to what a T cell requires for activation. Data on epitope expression levels revealed copy numbers between one and 10,000 per cell, although most lie between ten and 1,000 [12,38–49]. The epitope expression levels are likely to differ between different pathogens, depending on the genome size of the pathogen and on the absolute amount of pathogen protein that is produced in infected cells.

The relationship between T cell coexistence and the per-APC expression level of each epitope depends on the assumed size of the APC, on the number of different pathogen epitopes that are displayed on the surface of the APC, and on the sensitivity of T cells to epitope, i.e., the number of cognate ligands a T cell needs to interact with to become activated (Equations 1 and 2 and Figure 6A). Figure 6 shows how the relationship of T cell coexistence on the per-APC epitope expression level changes for increasing size of APCs (Figure 6B), increasing T cell sensitivity (Figure 6C), and increasing number of displayed epitope types (Figure 6D). In our simulations, APCs were assumed to be of a size such that six or ten T cells could interact simultaneously with each APC. However, especially dendritic cells (DCs), an important type of APCs, have a large surface area, which might allow for as many as 300 T cells to simultaneously interact with them [50]. Figure 6B shows that increasing the surface of an APC increases the region of epitope expression levels at which T cell coexistence is maximal. This is because competition for access to APCs is less severe when the surface area of an APC is larger. What happens if T cells are more sensitive to epitope, and require less than the 50 copies we assumed so far? Experimental studies observed that as few as one to ten copies of epitope might be sufficient to generate calcium fluxes (one of the earliest responses of T cells to antigen stimuli) in T cells [41,51–53]. When T cells are more sensitive to their cognate ligand, the T cell coexistence curve shifts to the left (Figure 6C). This is because epitope is less limiting when T cells require less epitope to become activated. As soon as epitope ceases to be limiting, access to the APC becomes limiting. Finally, when more different pathogen epitopes are displayed on the surface of the APC, this reduces the epitope expression level up to which T cell coexistence is maximal, but does not affect the epitope expression level at which the immunodominant T cell response outcompetes all other responses (Figure 6D). Competitive exclusion occurs when all sites on an APC are suitable for all T cell specificities (or at least for the dominant specificity), and is independent of the number of epitope types. However, maximal coexistence depends on each APC site only displaying one epitope type. Hence, the closer the number of epitope types to the number of T cell binding sites per APC, the sooner competition for access to APC sites sets in. Summarising, for APCs with a large cell surface, the range of epitope expression levels for which competition between T cells is for the specific epitope is quite wide. If T cells are very sensitive to their cognate ligand, this reduces the range of epitope expression levels for which T cells do not compete with each other.

**Figure 6:**
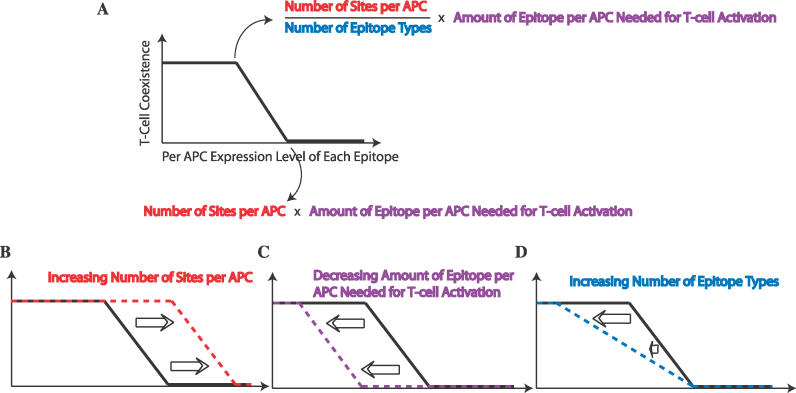
The Effect of Varying APC Size, T Cell Sensitivity, and the Diversity of Displayed Epitopes on T Cell Coexistence The per APC epitope expression level of each epitope up to which T cell coexistence is maximal depends on the size of APCs, the number of different epitope types that are presented, and the amount of epitope a T cell needs to become activated. The epitope expression level above which the immunodominant T cell population outcompetes all others solely depends on the APC size and on the amount of epitope required for T cell activation (A). In the three graphs below, the effect of changing any one of these parameters is shown. (B) The larger the surface area of APCs, the wider the range of per APC epitope expression levels for which coexistence of T cells is maximal. (C) The more sensitive T cells are to epitope (i.e*.*, the lower the amount of epitope a T cell requires to become activated), the more narrow the range of epitope expression levels with maximal coexistence of T cell populations of different epitope specificity. (D) The number of different pathogen epitopes that are presented on an APC effects the per APC epitope expression level up to which T cell coexistence is maximal, but not the level at which the immunodominant T cell specificity outcompetes the other epitope-specific T cell populations. The higher the number of different epitope types, the more narrow the range of per APC epitope expression levels at which T cell coexistence is maximal.

## Discussion

In this study, we investigated T cell competition and coexistence with an individual-based stochastic simulation model in which APCs have multiple T cell interaction sites, and addressed the following two questions: 1) Under what conditions do T cells specific for different epitopes of a pathogen compete with each other, and 2) why do some immunogenic epitopes fail to elicit a measurable T cell response? For the first question, the model predicts that, when epitope expression levels are low, competition is for the specific epitope, and hence immunodominant and subdominant T cells of different epitope specificity coexist readily. However, at high epitope expression levels, T cells compete for access to the APC, which is a shared resource. This competition can severely reduce the expansion of the subdominant T cell specificity, or even lead to the extinction of the subdominant T cell specificity (i.e., competitive exclusion). The second question, why only so few of all presented pathogen epitopes are targeted upon infection, has also been debated elsewhere [54,55]. The theoretical maximum breadth of T cell immune responses in our model is set by the maximum number of T cells that can simultaneously interact with one APC. Thus the size of an APC and the level at which epitopes are expressed, might explain why only a subset of the presented epitopes elicit measurable T cell immune responses.

The results shown in this paper are based on simulations in which epitope expression levels of the two epitopes are equal in amount and constant during the lifetime of the APC. Differential expression of epitopes [13,55], and the kinetics of epitope expression, can affect immunodominance [23]. Skewed epitope expression in favour of the low affinity T cell specificity by higher expression of the epitope can compensate partially or fully for the affinity disadvantage of the subdominant T cell response. On the other hand, a subdominant T cell specificity will be outcompeted more rapidly if the epitope of the immunodominant T cell specificity is overexpressed (unpublished data). Similarly, if the subdominant epitope is expressed earlier than the immunodominant one during the lifespan of an APC, this is expected to facilitate coexistence. Antia, et al. have argued that the lack of competition between T cell populations specific for different epitopes of a pathogen, such as observed in Vijh, et al. [24], may be due to the partly antigen-independent nature of T cell expansion [56]. In acute infection, when naive T cells are activated by their cognate epitope, antigen is usually abundant and hence T cells of different epitope specificity can expand independently of each other. Indeed, our model simulations show that increasing the rounds of programmed proliferation upon activation accelerates clearance of the pathogen in acute infection and leads to a slightly improved relative expansion of the subdominant versus the immunodominant T cell population than that shown in Figure 2A (unpublished data). However, the equilibrium T cell coexistence in chronic infection is robust to changes in the proliferative capacity of T cells (unpublished data). One other simplifying assumption in our model was that all APCs express the same amount of epitope. We checked for the effect of variability in epitope expression levels between APCs in our simulations and found that variability in epitope expression levels between APCs yields a moderate increase in the range of epitope expression levels at which T cells can coexist.

Our theoretical predictions offer potential explanations for some of the controversial results observed in T cell competition experiments. However, it remains problematic to compare different experimental systems directly. Firstly, the per-APC epitope expression levels are often not known. Secondly, the relationship between T cell coexistence and epitope expression levels is sensitive to factors such as the size of APCs, the sensitivity of the epitope specific T cells, and the number of different pathogen epitopes that are presented (see Figure 6). One example of controversial results are two adoptive transfer studies. In one, using OVA peptide specific OT-1 T cells, Kedl, et al. observed competitive interactions between T cells of different epitope specificity [18], whereas in another, using LCMV infection, Probst, et al. did not find evidence for competition [19]. Our theory would predict that epitope expression levels were low in the Probst study, and that therefore no competition was observed, whereas they were high in the Kedl study, leading to competitive interactions between the two T cell populations. However, part of the Kedl study was performed with DCs loaded with very low concentrations of peptide (approximately 10^−9^ M). So epitope expression levels alone cannot explain the discrepancy. Importantly, however, the OVA-specific T cells, used in the Kedl study, are known to be very sensitive to the OVA-peptide. Our model predicts that for highly sensitive T cells, competition between T cells of different epitope specificity already occurs at lower epitope expression levels, because access to APCs rather than epitope becomes limiting at lower epitope expression levels (see Figure 6C). Hence, it is conceivable that the high sensitivity of OVA-specific T cells leads to competition for access to APCs in the Kedl study, although the epitope expression levels in the Kedl and the Probst studies may have been similar.

The current view on T cell competition is that T cells of different epitope specificity can compete with each other when the ratio of T cells versus antigen is high and when epitopes are presented on shared APCs [14]. Our results suggest that, even when these prerequisites are met, T cells of different epitope specificity will only compete with each other when epitope is not limiting, i.e., when the per-APC epitope expression levels are high. Our model prediction could be tested experimentally by immunising mice with APCs loaded with high or low epitope expression levels. Our model predicts that immunodominance will be more pronounced at high than at low epitope expression levels.

## Materials and Methods

The code for our individual-based simulation was written in JAVA and is available upon request. Our IBM describes the dynamics of virally infected cells, APCs that display epitopes of the virus, and cytotoxic T cells that recognise these epitopes. Our model is based on that of De Boer, et al. [25], with one major difference: in De Boer's model, APCs consist of a set of independent sites that T cells can interact with to become activated. In our model, the sites on an APC are connected and thus not independent. This means that conjugation of a T cell with an APC site can affect availability of epitopes on adjacent sites.

### Time-step size and duration of simulations.

Each virally infected cell, APC, and T cell in our model is initialised with a certain set of specific properties. In each time step, a certain set of events (for example, T cell activation or death) can take place. The length of a time step is set to 1/50th of a day. This way, each event in the simulation occurs with only a small likelihood per time step, and hence the order in which methods are “called” in the simulation is irrelevant. All simulations last 400 days (20,000 time steps). For most parameter settings, the simulations equilibrate within that timespan. The equilibria in our simulations are either stable steady states or stable oscillations, or, in some cases, slow transients. In any case, simulations are stopped after 400 days. All rates (time-dependent parameters) were scaled by the time-step size at initialization of the simulations, i.e., converted into per-time-step probabilities.

### Dynamics of virally infected cells.

We set the probability of proliferation of T cells and the rate of clearance of infected cells by T cells such that the virus is not eradicated by the T cell response, but causes a chronic infection. Viral replication is assumed to be density dependent, i.e., in the absence of a T cell response, the population of virus-infected cells grows to its carrying capacity, which is set to 10^5^. For the sake of simplicity, we do not explicitly model free viral particles and target cells, but only virally infected cells.

Virally infected cells are cleared by T cells at a probability given by the product of the rate of clearance of infected cells, which is set to 10^−5^, and the number of effector T cells (see the next section, APCs). Although we refer to the pathogen as a viral pathogen throughout this chapter, the model can be applied to any pathogen that is displayed on MHC class I molecules on APCs, including intracellular bacteria or cross-presented extracellular pathogens (see, e.g., reviews [57,58]). We chose not to include the T-helper cell populations in our model, because this would add an additional layer of complexity and because T cell competition experiments that use epitope-loaded, mature DCs as antigen are also conducted in the absence of T-helper cells.

### APCs.

The biology of epitope presentation on MHC class I molecules in infected APCs is complex. In our model, we implement antigen presentation heuristically. We assume that the number of infected APCs is proportional to the number of virally infected cells, and set the number of new epitope-presenting APCs per day to be the product of the infected cells and a constant, *σ*. The APCs in our model have multiple T cell binding sites, and the default number of T cell binding sites per APC is set to six. Recent imaging studies have observed one to 14 T cells interacting simultaneously with one DC, which is an important type of APC [59,60]. However, the total surface area of DCs might be large enough to allow for up to 300 T cells to interact with one DC simultaneously [50].

In our model, we assume that a T cell needs to interact with 50 copies of its specific epitope–MHC complex to form a conjugate with an APC, and that each APC expresses 300 copies of each of the two epitopes. Throughout this paper, the per-APC epitope expression level is varied and indicated at each simulation result. Quantitative data on epitope expression levels on APCs in the literature suggest values ranging from one to 10,000 copies per epitope [12,45–49]. The minimal number of epitope ligands needed to activate a T cell is very low, and may be less than ten copies [53,61]. For our model studies, only the ratio of the epitope expression level per APC and the number of epitope copies required to activate a T cell is relevant. This ratio needs to be larger than or equal to one to allow one or more T cells of a particular specificity to interact and potentially become activated by an APC. In our model, epitopes are assumed to move freely on the surface of APCs, and, when a T cell forms a conjugate with an APC site, the amount of epitope the T cell needs to become activated is allocated to this site. For simplicity, the two foreign epitopes modelled here are assumed to be presented in equal densities on the APC. Effects of unequal presentation densities are discussed under Discussion.

### APC death.

In vitro data on the survival of DCs suggests that they live on average for two to six days [62,63]. In our model, we set the average lifespan of APCs to five days (the per-day probability of death of an APC is set to 0.2). When an APC dies, the T cells that are conjugated to it dissociate without becoming activated. We have not included the killing of APCs by T cells, since including APC killing by T cells does not alter the results qualitatively (unpublished data).

### T cells.

Naïve T cell precursor frequencies have been estimated to be approximately one in 10^5^ cells, that is, 10 to 100 precursor cells per specificity per mouse [64–66]. At the start of a simulation, we seed small populations of two types of epitope-specific T cells (50 cells each) into the model immune system. The two T cell specificities can be distinguished by their T cell receptor specificity (set to 0 or 1), and only differ from each other in terms of their probability of proliferation upon dissociation from a conjugate with an APC site. In our model, the probability of proliferation is a composite parameter that describes the intensity of signals a T cell receives while it is in conjugation with an APC site. This signal strength is shaped, among other things, by the TCR density on the T cell membrane, the affinity of a TCR to its cognate epitope, and the density of the cognate epitopes on the APC surface (the latter is set equal for both epitopes). For simplicity, we will refer to T cells with a high probability of proliferation as immunodominant or high-affinity T cells and to those with a low probability of proliferation as subdominant or low-affinity T cells. T cells occur in the following states: as effector T cells, conjugated with an APC site, or proliferating.

### “Scanning” and conjugate formation.

In the model, there are two kinds of effector T cells: “scanning” effectors and “pure” effectors. A scanning effector T cell scans 50 randomly picked APCs with free binding sites for antigenic stimuli per day and, upon success, it engages in a conjugate with the binding site. We assume that T cells only scan APCs with one or more free binding sites. This is because APCs that are fully covered with T cells are no longer accessible to T cells. Most data on T cell and DC interaction rates count the number of T cells that scan one DC per hour when antigen is absent. These estimates range from 500-5000 per hour [50,59,60,67,68]. For a T cell precursor frequency of one in 10^5^ cells, this would mean that a DC encounters a T cell specific for a particular epitope once every 20–200 hours (i.e., approximately one to ten per day). For the situation when a specific antigen is present, and specific T cell levels are far higher than precursor levels, the contact rate is naturally much higher, motivating us to set the number of APCs that a T cell scans per day to 50. In equilibrium, APCs with free binding sites are very rare. If all APCs are full, scanning of APCs by T cells stops until some sites become free again. Once a T cell has conjugated with a site on an APC, it remains bound to the APC site for an average of one day. Upon dissociation from the conjugate, the T cell proliferates according to its probability of proliferation (also referred to as “T cell affinity”).

Experimental data on the dynamics of the interactions between APCs and T cells suggests that T cells first engage in multiple, short encounters, then in longer interactions that last up to 30 minutes [69]. For our model results, both kinds of interactions are qualitatively equal. What matters is the total interaction time, during which a T cell occupies space and/or epitopes on an APC. Hence, we have condensed the multiple conjugate interactions into one long-lasting interaction of an average of one day.

### Proliferation.

T cells become activated by interacting with an APC site that presents its specific epitope. The probability that a T cell starts to proliferate upon dissociation of the T cell–APC conjugate is varied in the simulations, but usually set to values around 0.1. Recently, it was shown that T cells, after an initial lag-phase of approximately one day, go through five to ten rounds of proliferation without the need for reexposure to the antigen [70–73]. Previously, it was assumed that T cells require antigen contact for each round of expansion. This antigen-independent sequence of several rounds of division upon activation is referred to as programmed proliferation [74]. In our model, activation of the T cell upon dissociation from the APC immediately leads to the first of *n* rounds of programmed proliferation. The subsequent rounds of proliferation are more rapid, and occur with a rate of three per day. In our model, the default number of rounds of programmed proliferation of an activated T cell is set to five. In our model, we do not consider clonal exhaustion [75]. Hence, if antigen is not cleared, epitope-specific T cell populations can theoretically live indefinitely. Still, because of their high death rate, individual T cells typically only become activated and go through programmed proliferation once or twice during their lifespan (see section on T cell mortality further on).

### Effector function.

After *n* rounds, proliferation stops and the T cells become “pure” effector cells. Effector T cells clear virally infected cells, and do not scan APCs for further antigenic stimulation. T cells remain in the pure effector state for an average of five days and subsequently start scanning APCs again, in search of antigenic stimuli. Scanning T cells continue to clear virally infected cells until they engage a new conjugate with an epitope-presenting T cell binding site on an APC. For simplicity, we assume that there is no maximum to the number of times that T cells can become activated and can undergo programmed proliferation.

### T cell mortality.

Model-assisted data analysis has estimated mortality rates for T cells between 0.05 and 0.2 per day [76,77]. In our model, we set the rate of death of T cells to 0.2 per day. We further assume that T cells do not die during programmed proliferation.

### Parameter values.

The parameter values used in our model simulations are, where possible, based on experimental values (see Table 1). Since it is our objective to study under what circumstances T cell populations of different epitope specificity are in competition with each other, we look at longer-term interactions of T cell populations and their resource, and hence evaluate equilibrium results of chronic infections. When T cells are stimulated very efficiently by the antigen, or if they have a very high viral killing rate, they can rapidly clear infections. In our model, efficient stimulation of T cells can arise from, for example, high probabilities of proliferation of T cells, a high number of rounds of proliferation upon activation, or very efficient antigen presentation by infected APCs (i.e*.*, high *σ*).

## Abbreviations

APC: antigen-presenting cell
DC: dendritic cell
IBM: individual-based model
MHC: major histocompatibility complex
